# Metabolic Profiling and Post-harvest Behavior of “Dottato” Fig (*Ficus carica* L.) Fruit Covered With an Edible Coating From *O. ficus-indica*

**DOI:** 10.3389/fpls.2018.01321

**Published:** 2018-09-05

**Authors:** Alessio Allegra, Alessandra Gallotta, Francesco Carimi, Francesco Mercati, Paolo Inglese, Federico Martinelli

**Affiliations:** ^1^Department of Agricultural, Food and Forest Sciences – Università degli Studi di Palermo, Palermo, Italy; ^2^Department of Soil, Plants and Food Science (DiSSPA), University of Bari, Bari, Italy; ^3^Institute of Biosciences and BioResources, Division of Palermo, National Research Council, Palermo, Italy

**Keywords:** amino acids, edible coating, fig, fruit quality, metabolism, metabolomics

## Abstract

Fig fruits are usually highly sensitive to some physiopathological disorders during post-harvest life, such as softening and skin cracking. Indeed, the use of edible coating (EC) has been evaluated in several fruit crops to reduce fruit post-harvest transpiration and to maintain fruit visual quality. The aim of this study was to determine the post-harvest metabolic response of breba figs treated with mucilage extract from *O puntia ficus-indica* cladodes, using an untargeted metabolomic approach. Coated and non-coated (control) fruit were sealed in plastic bags, and stored at 4°C for 7 days. The effect of the ECs on their quality fruit during cold storage and qualitative attributes were evaluated by analyzing the fruit primary metabolism and other qualitative parameters such as total soluble solids (TSS) content, titratable acidity (TA), fresh weight loss and firmness. Results underlined that EC was effective in maintaining fruit fresh weight, and fruit firmness. Stepwise discriminant analysis was able to discriminate fruit conditions. Alanine, xylulose, aspartic acid, glutamic, acid and 2,5-dihydroxypyrazine showed a significant role on discriminating edible coated fruit from untreated ones. Principal component analysis (PCA) was able to highlight clear differences in the overall metabolism changes between untreated and treated fruit. The application of EC significantly mitigated the decrease of most of the aminoacid content during cold storage. EC treatment caused the changes of several organic acids in comparison to untreated control, increasing the amount of carbohydrates and other key metabolites, such as beta-sitosterol, glycerol, and uracil. These results clearly showed the drastic effects of EC on fig metabolism during post-harvest and shed light on the beneficial mechanisms of this treatment.

## Introduction

Fig (*Ficus carica* L.) is native to western Asia and has been cultivated and consumed in the Mediterranean Basin since the earliest stages of the agricultural civilization (Accademia dei Georgofili, 2003). Indeed, Turkey, Egypt, Algeria, and Morocco account for 65% of the world production (FAOSTAT, 2015)^1^. Turkey is the leading Country with 27% of world fresh figs and 53% of dry figs, accounting for 51% of fig fruit world exports (Yilmaz et al., 2017). The common fig is a woody perennial species with a very large genetic base. Most of the fig genotypes produce parthenocarpic fruit, while some types produce syconia with only female flowers that will develop into the edible seeded figs (Stover et al., 2007). Common type figs are able to produce one (unifera types) or two crops (bifera types). “Dottato” fig is an ancient bifera type cultivated variety originated in Italy and most important in Southern Italy, particularly in the areas of Cilento, Calabria, and Sicily. It can produce two crops; the first crop (brebas) is parthenocarpic and fruit are named as brebas, while the second crop produces seeded figs only after pollination. Fruit ripening o the different genotypes ranges from the middle of June to late October. In Italy, breba figs are harvested from the end of June to the beginning of July, while “Forniti” (name for the second harvest) are harvested from early August to late September (Allegra et al., 2017). Both brebas and common figs are climacteric fruits and are slightly sensitive to high temperature on stimulating softening and decay severity. Moreover fresh fig quality is highest at full ripeness, but such soft figs are especially sensitive to damage. The type and degree of skin damage varied among cultivars (Kong et al., 2013). Consumers prefer fresh figs at the tree ripe maturity stage (Crisosto et al., 2010), color of skin and genotype (Çalişkan and Polat, 2011) but figs are highly susceptible to postharvest deterioration (Türk, 1989). The fig “Fioroni” have an higher epidermis perishability and a lower sugar content compared to fig “Forniti” (Kaynak et al., 1997). Fig fruit particularly sensitive to softening, skin cracking and, thus, to a severe post-harvest decay (Crane, 1986; Crisosto and Kader, 2004; Kong et al., 2013). The use of an edible coating (EC) extracted from cladodes of *Opuntia ficus-indica* (OFI) (Medina-Torres et al., 2000) was recently investigated on breba fig fruit (Allegra et al., 2017). EC reduced water transpiration, maintained fruit fresh weight, visual score values, fruit firmness and total carotenoid content, resulting in a longer cold storage (Allegra et al., 2017). Usually, studies of ECs applied to extend fruit shelf life are based on chemical and physical parameters (Guilbert et al., 1996), but no information is available on the metabolites involved in fruit growth and ripening with and without EC. Metabolomics is a powerful instrument that allows analyzing and quantifying simultaneously 100s of metabolites, linking them to transcriptomic and proteomic data. Metabolic approach is usually seen as complement to transcriptomics and proteomics because metabolic reactions are usually modulated not only by transcript and protein abundance but also by post-transcriptional and post-translational mechanisms (Rizzini et al., 2010; Martinelli et al., 2015). GC-MS is the common method that allows analyzing small molecules belonging the primary metabolism (carbohydrates, amino acids, and organic acids). Metabolomics allow to perform untargeted analysis and discover metabolic biomarkers linked with early stage of particular phenotypes and/or response to treatments (Johnson et al., 2016). The identification of biomarkers linked with physiopathological stages is a is a crucial challenge (Bundy et al., 2009) allowing to early start therapeutic measures and revert the stress condition to an healthy status. Metabolome approach is extremely useful in plant sciences since metabolites influence the plant phenotype more directly than transcripts or proteins (Saia et al., 2015). Metabolites are the final results of the different regulation levels occurring in cells and tissues. Metabolic profiling and non-targeted metabolomic approaches have recently been exploited to understand metabolic changes and identify biomarkers in fruit, in relation to agronomic treatments and biotic/abiotic stresses (Tosetti et al., 2012; Martinelli et al., 2013; Ibanez et al., 2014). The combination of transcriptome sequencing and metabolomics will allow identifying key points and important stress-related proteins in crops such as dehydrins (Natali et al., 2007). This integrated metabolomic approach will allow gain insight into the molecular regulation mechanisms of key metabolites at nutritional and qualitative perspectives of fruits and vegetables under post-harvest stage.

The analysis of the primary metabolism is essential for the qualitative characterization of the fruits after the post-harvest treatments. Primary metabolites are essential determinants of fruit quality. Although EC might affect the phenylpropanoid and carotenoid pathways, more changes are expected on the primary metabolism. It is well-known that the contents of primary metabolites are subjected to significant changes during post-harvest stage. The abundance of a particular metabolite can show either similarities or differences between different post-harvest treatments. The respiration process of the fruit is mainly affecting primary metabolism and this process is usually inversely associated with storability of post-harvest fruits. Indeed the wide analysis of primary metabolism can help to characterize the shelf-life after post-harvest or quality of fruits during cold storage. This would allow to identify key metabolic markers of fruit physiopathological conditions. Amino acids, carbohydrates, organic acids, and lipids are also essential parameters to be analyzed because they are mostly responsible of modifications of technological and nutritional parameters. Metabolomic approach with GC-MS is the best way to perform a wide, untargeted analysis of 100s of metabolites. This method allows to determine relative quantitative changes of primary metabolites in response to different treatments such as post-harvest conditions. Basing on these considerations, the objectives of this study were to evaluate: (1) the fruit characteristics and metabolomics changes of breba figs during post-harvest in response to coating treatments with edible mucilage obtained from *O. ficus-indica* cladodes (Matsuhiro et al., 2006) and (2) link changes in key metabolites with technological and physiological measurements.

## Materials and Methods

### Plant Materials

Breba fig (*Ficus carica* L.) fruits (yield/tree 52 ± 5.0 kg), cv. “Dottato,” were sampled at commercial harvest date (Crane, 1986) from six trees grown in a commercial orchard. A total of 54 fruits were randomly harvested when the average fruit weight and firmness were, respectively, 78 ± 2.1 g fw and 24.8 ± 2.3 N. After harvest, fruits were immediately dipped in chlorinated water (100 ppm of free chlorine) for 6 min and placed in bi-oriented polystyrene (PS) macroperforated bags (Carton Pack s.r.l., Rutigliano, Italy) used in another work on the Breba fig (Allegra and Colelli, 2017) Eighteen fruits were treated with a mucilage coating solution (MC), extracted from OFI (Sepulveda et al., 2007; Allegra et al., 2016, 2017), for 60 s; the exceeding coating was drained and the coated figs were dried in a forced-air dryer (20°C) for 30 min. Eighteen fruits were dipped in distilled water and used as a blank (CTR). Successively, in order to evaluate the quality fruit storage after treatment, MC and CTR samples were stored at 4 ± 0.5°C and 85% RH for 7 days while other eighteen fruits were analyzed immediately after commercial harvest time without treatment (T), and used as reference. Fruit fresh weight, firmness, total soluble solid (TSS) and titratable acidity (TA) contents were analyzed as described in Allegra et al. (2017).

### Metabolite Extraction and Derivatization

The lyophilized extracts of five biological replicates from each thesis (MC, CTR, and T) were analyzed at metabolomic level. Two milliliters of pre-chilled extraction solvent (MetOH:CHCl3:1:1) (v/v) were added to 20 mg of ground tissues (mesocarp and epicarp), incubated at 4°C with gently stirring for 5 min and then centrifuged at 6000 rpm for 2 min. Twenty microliters of supernatant was completely dried in a SpeedVac concentrator. Derivatization was performed with methoxyamine and N-methy-N-(trimethylsilyl) trifluoroacetamide and separations were conducted with an Agilent 6890 GCgas chromatographer (GC) coupled to a quadrupole mass spectrometer (MS) and equipped with a Zorbax-C 8 column. Samples were analyzed in both splitless and split modes. All the conditions of mass spectrometry dealing with splitless and split conditions, the source temperature and temperature increase and all other parameters were used as described by Martinelli et al. (2013). The MS was set at 150°C, with a 5.90 min solvent delay time and scan range from 50 to 600 atomic mass units. Prior to the acquisition, the MS was autotuned using FC43 according to the instrument manual.

### Data Acquisition and Peak Identification

The relative concentrations of each metabolite were determined by peak areas. Metabolite identification was performed using the Agilent Fiehn GC/MS Metabolomics RTL Library containing more than 700 common identified metabolites in relation to their GC/MS EI spectra and retention time indexes. Subsequently, all peak detections were manually checked for false positive and false negative assignments and corrected with retention time locking to reduce run-to-run retention time variation.

### Statistical Analysis

Significant differences in the metabolic profile among thesis investigated (MC, CTR, and T) were evaluated through one-way ANOVA using Systat 13.2 (Systat Software Inc.), followed by Fisher’s test (*P* ≤ 0.05). Selected compounds showing significant difference were grouped in four main classes of metabolites and their relative abundance among analyzed samples were illustrated through heatmap clustering by using the R package ggplot2 (Wickham, 2016). To reduce complexity and runtime, a first selection of variables able to discriminate the treatments was carried out through SDA (Stepwise Discriminant Analyses) using SYSTAT 13.2 (Systat Software Inc.). SDA was performed both among characterized classes and on the overall significant data collected. Subsequently, a principal component analysis (PCA) on selected panel was run using the R package FactoMiner (Le et al., 2008). PCA enabled us to clear assess the effect of selected variables on the treatment discrimination, extracting the main orthogonal variables explaining the divergence among thesis. Before carrying out SDA and PCA analysis, mean values per trait were standardized to a mean equal to 0 and standard deviation equal to 1. Finally, the Pearson correlation coefficient (*p* < 0.05) was calculated between the profiles of all pairs of selected metabolites using Hmisc R/package^2^ and a scatter plot with the correlation coefficients and their significance was developed with PerformanceAnalytic R/package^3^.

## Results

### Quality of Breba Fig Fruit During Storage

TSS and TA contents in breba fig fruit did not change after treatments. Brebas showed a significant (*p* < 0.05) decrease in firmness during storage, significantly influenced by the coating treatment. Indeed, MC retained 75% of the initial firmness comparing to commercial harvest time (T), while fruit firmness of CTR was 45% of T and 25% less than MC fruit at the same sampling date (**Table 1**). MC fruit did not show any significant weight loss, while CTR showed a significant decrease in fresh weight in comparison to T (**Table 1**).

**Table 1:** Total soluble solid and titratable acidity contents, firmness and weight loss of breba fig fruit (*Ficus carica* L.) at commercial harvest time (T), coated with *O. ficus-indica* (OFI) mucilage (MC) and untreated (CTR) after 7 days at 4°C.

	Fruit stored after 7 days at 4°C storage		
	Commercial harvest time (T)	Untreated (CTR)	Treated with edible coating (MC)
Total soluble solid (°Brix)	16.8 ns	16.3	16.9
Titratable acidity (g L^-1^)	0.8 ns	0.8	0.7
Firmness (N)	24.8 a	13.9 c	18.7 b
Weight loss (%)	a^∗^	2.56 b	0.65 a

*Data are means n = 9. Different letters indicate significant differences between treatments (Fisher’s exact test, p ≤ 0.05); ns = non-significant differences. ^∗^Reference value of fresh weight at commercial harvest time was 78 g.*

### Metabolite Profiling Analysis

The non-targeted metabolomic analysis displayed a total of 176 peaks, 112 of which were identified though a comparison with the Agilent Fiehn GC/MS Metabolomics RTL Library and mass spectra interpretation (**Supplementary Table S1**). The compounds identified were grouped in four classes: (1) Amino acids, (2) Carbohydrates, (3) Organic Acids, and (4) Others. Seven days after storage CTR showed a significant reduction of all 15 amino acids or amino acid-derivatives (**Table 2**). On the contrary, treatment with mucilage EC significantly reduced this detrimental effect in MC. All amino acids or their derivative were higher in MC treatment compared to CTR at 7 days after storage except for beta-alanine and oxoproline. While tryptophan, asparagine, alpha-ketoglutarate, and cyanoalanine amount in MC was similar to harvest time (T) (**Table 2**).

**Table 2:** Different Metabolites (groups of amino acids or their derivative) (expressed as area, in arbitrary units, of chromatographic peaks) of breba fig fruit (*Ficus carica* L.) at commercial harvest time (T), coated with *O. ficus-indica* (OFI) mucilage (MC), and untreated (CTR) after 7 days at 4°C.

	Commercial harvest time (T)	Untreated (CTR)	Treated with edible coating (MC)
Tryptophan	8904 a	1742.6 b	7027.8 a
Threonine	11986.8 a	4102.6 c	6695.0 b
Phenylalanine	4930.8 a	2567.2 c	3782.8 b
Oxoproline	38778.8 a	20912.2 b	17818.8 c
Isoleucine	28903.6 a	13485.4 c	21922.0 b
Glutamine	33219.2 a	6960.2 c	14649.0 b
Aspartic acid	21718.8 b	11552.6 c	30791.6 a
Valine	51660.2 a	22802.6 c	32661.8 b
Asparagine	149836.4 a	50307.6 b	152507.2 a
Alanine	326458.0 a	116876.4 c	243384.2 b
Serine	68068.8 a	29507.0 c	41905.4 b
Beta-alanine	507.2 a	350.0 b	305.0 b
Alpha-ketoglutarate	333.6 a	235.6 b	398.4 a
O-acetylserine	853.2 a	221.6 b	358.2 b
Cyanoalanine	4942.2 a	2125.6 b	5592.8 a

*Data are means n = 5. Different letters indicate significant differences between treatments (Fisher’s exact test, p ≤ 0.05); ns = non-significant differences.*

The MC treatment caused also the changes of several identified organic acids in comparison to CTR (**Table 3**). In fact, after 7 days the content of different organic acids, such as succinic acid, saccharic acid, lactic acid, gluconic acid, pipecolic acid, ribonic acid, benzoic acid, 2-hydroxyglutarci acid, stearic acid and linoleic acid was reduced in CTR in comparison to T. On the other hand, treatment with OFI extract increased significantly the concentration of glutamic acid, citric acid, malic acid, pipecolic acid, benzoic acid, malonic acid, pelargonic acid, stearic acid, and linoleic acid in comparison to CTR. Interestingly, glutamic acid, malic acid, malonic acid, pelargonic acid, stearic acid, and linoleic acid were higher in MC than reference (T) analyzed at commercial harvest time.

**Table 3:** Different Metabolites (groups of Organic acid) (expressed as area, in arbitrary units, of chromatographic peaks) of breba fig fruit (*Ficus carica* L.) at commercial harvest time (T), coated with *O. ficus-indica* (OFI) mucilage (MC) and untreated (CTR) after 7 days at 4°C.

	Commercial harvest time (T)	Untreated (CTR)	Treated with edible coating (MC)
Succinic acid	4039.4 a	1171.8 b	725.0 c
Saccharic acid	2087.2 a	1311.8 b	1777.0 b
Lactic acid	31640.8 a	1041.6 b	1137.4 b
Isothreonic acid	1252.0 b	1345.2 a	663.0 c
Glyceric acid	14161.6 a	14490.8 a	8014.0 b
Glutamic acid	5191.4 b	4414.2 b	7389.8 a
Glycolic acid	1266.2 a	383.6 c	453.6b
Citric acid	345383.8 b	528503.2 b	716363.0 a
Gluconic acid	199599.4 a	2978.6 b	3241.4 b
Malic acid	74869.6 b	62302.2 b	135343.2 a
Pipecolic acid	81100.0 a	46366.4 b	90840.0 a
Ribonic acid	654.2 a	392.4 b	508.8 b
Benzoic acid	904.2 a	793.8 b	1047.0 a
2-hydroxyglutaric acid	469.0 a	205.2 b	241.8 b
Malonic acid	238.2 b	222.4 b	451.8 a
Pelargonic acid	998.0 b	788.4 b	1113.4 a
Stearic acid	16771.4 b	13896.0 c	19832.8 a
Linoleic acid	321.6 a	149.6 b	387.2 a

*Data are means n = 5. Different letters indicate significant differences between treatments (Fisher’s exact test, p ≤ 0.05); ns = non-significant differences.*

Although fewer differences were highlighted for carbohydrates, at 7 days post-harvest storage we observed a significant reduction of several metabolites in CTR (**Table 4**). The content of, xylonolactone, inositol-4-monophosphate, galactinol and xylulose was reduced in CTR comparing to the commercial harvest time (T). On the contrary, we observed a significant increase of glucose-6-phosphate, fucose, sucrose-6-phosphate, xylonolactone, erythronic acid lactone, hexose-6-phosphate, and fructose-6-phosphate in MC compared to both CTR and T.

**Table 4:** Different Metabolites (groups of Carbohydrates) (expressed as area, in arbitrary units, of chromatographic peaks) significantly different in of breba fig fruit (*Ficus carica* L.) at commercial harvest time (T), coated with *O. ficus-indica* (OFI) mucilage (MC) and untreated after (CTR) 7 days at 4°C.

	Commercial harvest time (T)	Untreated (CTR)	Treated with edible coating (MC)
Ribose	7572.4 a	6754.0 a	3192.2 b
Isomaltose	1693.0 b	1796.6 a	1576.8 c
Glucose-6-phosphate	1051.0 b	993.2 b	2699.0 a
Fucose	4373.0 bc	3470.6 c	7443.0 a
Erythritol	2968.4 a	3184.2 a	618.2 b
Threitol	590.2 b	1123.6 a	701.0 b
Sucrose-6-phosphate	292.8 b	203.0 b	429.6 a
Xylonolactone NIST	1301.4 b	869.6 c	1620.0 a
Ribitol	891.2 a	554.0 a	756.8 a
Inositol-4-monophosphate	154.6 a	85.4 b	217.0 a
Erythronic acid lactone	205.6 b	207.2 b	331.0 a
Galactinol	1269.4 a	703.0 b	774.2 b
Xylulose NIST	4120.2 a	1430.6 b	3754.6 a
Hexose-6-phosphate	364.4 b	301.2 b	902.8 a
Fructose-6-phosphate	643.6 b	421.0 b	998.2 a

*Data are means n = 5. Different letters indicate significant differences between treatments (Fisher’s exact test, p ≤ 0.05); ns = non-significant differences.*

Finally, other metabolites (beta-sitosterol, glycerol, uracil, 5-hydroxynorvaline, and phenol) showed significantly increase in the coated fruit (**Table 5**).

**Table 5:** Other Metabolites (expressed as area, in arbitrary units, of chromatographic peaks) significantly different in commercial harvest time (T), coated with *O. ficus-indica* (OFI) mucilage (MC) and untreated (CTR) after 7 days at 4°C.

	Commercial harvest time (T)	Untreated (CTR)	Treated with edible coating (MC)
Phosphate	25350.6 a	15493.0 c	19971.6 b
Epsilon-caprolactam	959.4 b	784.8 c	1262.2 a
Beta-sitosterol	6111.4 b	3650.4 c	7632.6 a
Glycerol	33722.4 b	5399.4 c	136120.0 a
Urea	2265.2 a	912.6 b	898.6 b
Uracil	300.8 a	188.4 b	225.2 a
Alpha-aminoadipic acid	1340.6 a	481.8 b	512.4 b
5-hydroxynorvaline NIST	427.2 a	285.4 b	521.8 a
2.5-dihydroxypyrazine NIST	425.2 a	265.4 b	559.4 a
Phenol	536.4 b	513.4 b	787.8 a

*Data are means n = 5. Different letters indicate significant differences between treatments (Fisher’s exact test, p ≤ 0.05); ns = non-significant differences.*

### Functional and Statistical Data Analysis

Selected metabolites with significant differences among treatments (58), belonging to amino acids or their derivative (15 metabolites), carbohydrates (15), organic acids (18), and other metabolites (10) were illustrated through heatmap clustering and used in the SDA and PCA analysis.

**FIGURE 1:**
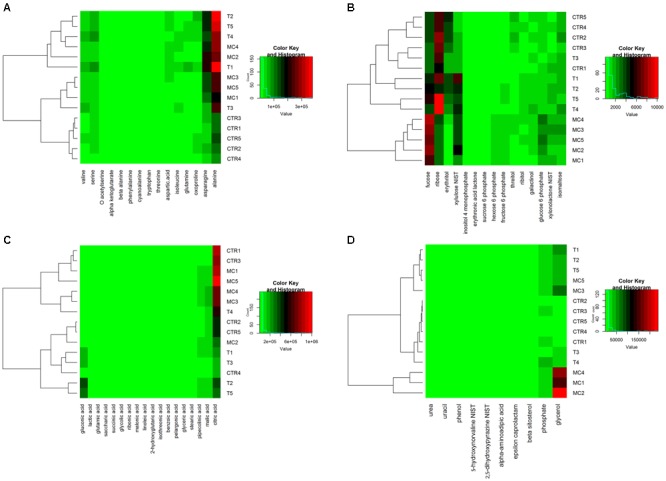
Heatmap representation of metabolic profiles of MC (fruits treated with mucilage coating after storage), T (reference fruits after harvest), CTR (blank after storage) for each identified class of compounds: **(A)** amino acids; **(B)** carbohydrates; **(C)** organic acids; and **(D)** other metabolites. Green and red colors represent reduced and augmented representation levels, respectively. A hierarchical clustering of samples is also shown.

In order to obtain a simplified representation of the quantitative behavior of selected metabolites among treatments, a heatmap clustering for each class of compounds was generated (**Figure 1**). Among classes, amino acids (**Figure 1A**) with significant differences grouped only the samples without treatment (CTR) in a private cluster with a low amount of alanine and asparagine, comparing to T and MC fruits. On the contrary, selected carbohydrates (**Figure 1B**) allowed discriminating the different treatments except for samples CTR1 and T3, with fucose and ribose highly represented in MC and T/CTR, respectively. In the organic acids and other metabolites classes (**Figures 1C,D**), selected metabolites didn’t allow to clustering the different thesis, except glycerol that grouped three out of five samples belonging to MC.

**FIGURE 2:**
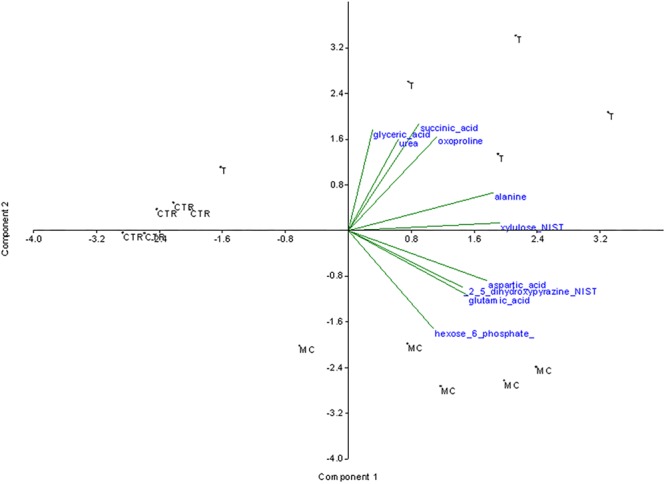
Stepwise discriminant analysis of the 58 significantly variable metabolites among the three treatments. The metabolites mostly contributing to the separation of the treatments were indicated. MC (fruits treated with mucilage coating after storage), T (reference fruits after harvest), CTR (blank after storage).

Unlike the PCA, the SDA takes into account the information contained in the classes and aims to identify a subset of metabolites which most discriminate against the treatments compared. Therefore, the initial selection of variables was carried out by performing SDA on a selection of variables (**Figure 2**). The second component seems to discriminate the observations of T and MC from CTR. The second component instead seems to differentiate the observations of CTR from MC and T. The following metabolites weigh more and positively on the first component: alanine, xylulose and aspartic acid, glutamic acid and 2_5_dihydroxypyrazine. Therefore the observations of the T and MC treatments should be characterized by higher mean values these variables with respect to the CTR observations. Succinic acid and glyceric acid (followed by oxoproline and urea) contribute positively more on the second component while hexose-6-phosphate negatively contribute to the same component. T treatment is therefore characterized by the presence of higher average values compared to MC for the first group of components while it is lower than MC regarding hexose-6-phosphate.

The ANOVA was performed using all the 112 metabolites. Of them, only 54 resulted to have significantly changes among treatments. SDA was performed using these significantly changing metabolites. The SDA conducted allowed to select the following 10 variables that mostly contribute to the data variance: alanine, aspartic acid, oxoproline (amino acids), hexose_6_phosphate, xylulose (carbohydrates), succinic_acid, glutamic acid, glyceric acid (organic acids), and 2,5-dihydroxypyrazine, urea (other metabolites).

**FIGURE 3:**
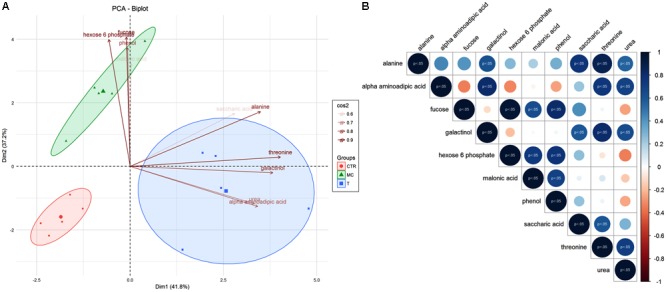
**(A)** Principal Component Analysis (PCA) of selected metabolites for T (reference fruits after harvest), MC (fruits treated with mucilage coating after storage), CTR (blank after storage). Metabolites associated with separation of treatments were indicated in the plot, underlining their significance values (0.6 < cos2 < 0.9). **(B)** Person correlation matrix of 10 selected metabolites correlated to MC, T, and CTR separation. Positive correlations are displayed in blue and negative correlations in red color. Color intensity and the size of the circle are proportional to the correlation coefficients. The significant correlations (*p* < 0.05) were highlighted.

Some of selected variables were able to discriminate all investigated treatments (T, CTR, and MC). Indeed, in PCA analysis the first component was able to discriminate reference fruit (T) from CTR and MC samples, while the second one allowed differentiating MC from CTR and T (**Figure 3A**). Fucose and hexose-6-phosphate, phenol were the most weighing for Dim1 (**Figure 3A**) while alanine, threonine, galactinol, urea, alpha-aminoadipic acid mostly contributed to the variability explained by Dim2 (**Figure 3A**). Seventy-nine percent of the total variance was explained by the two first components, with alanine, threonine, fucose, and hexose-6-phosphate as the main variables (cos2 ≥ 0.9) defining the divergence among thesis. These evidences were also in agreement with correlation analysis (**Figure 3B**). In fact, Pearson correlations were computed for the 10 variables selected and only positive correlation between metabolite levels was significant (*p* < 0.05). Among these alanine VS threonine and fucose VS hexose-6-phosphate showed the higher correlation coefficients, 0.87 and 0.96, respectively (**Figure 3B** and **Supplementary Figure S1**).

## Discussion

The aim of this study was to link fruit technological and physiological parameters with key metabolite changes mainly of the primary metabolism in breba fig during post-harvest in response to coating treatment. PCA based on different categories of metabolites (amino acids, carbohydrates, and organic acids) was effective in clearly distinguishing fruit at commercial harvest (T) from those maintained 7 days at 4°C during post-harvest without any treatment (CTR) and those treated with edible coating (MC). These results highlighted that EC have profound effects on the amount of metabolites belonging to primary metabolism. In addition these data pointed out how MC may have a positive effect on the maintaining of high quantitative levels of amino acids, carbohydrates and several important organic acids. PCA developed by using the carbohydrates class, summarizes the contribution of different variables identified related to the samples analyzed. Indeed, the main two components explained 79% of total variability. The samples were clearly grouped based on their treatment (T, CTR, and MC). Interestingly, there is a robust correlation between: (a) T and threonine, galactinol compounds and (b) MC to glucose/fructose-6-phosphate and fucose that are the two main variables with strong weight.

The synthesis of galactinol and raffinose family oligosaccharides (RFOs) has been reported in vegetative tissues in response to a range of abiotic stresses, such as drought (Dos Santos et al., 2015), therefore galactinol and raffinose may function as osmoprotectants in drought-stress tolerance of plants (Taji et al., 2002). In addition, RFOs have an important role in the carbohydrates storage for many plants (Sprenger and Keller, 2000). Since the biosynthetic pathways of the substrates of hemicellulose and pectins (e.g., xylose, arabinose, galactose, rhamnose, galacturonic acid, and fucose) may be sustained by monomers availability also from galactinol and raffinose transformation; the galactinol compounds found in T could enhance the pectin development.

Pectin is a main component of the plant cell wall and is the most complex family of polysaccharides in nature, such as xylose, galactose, fucose and more (Liepman et al., 2010). The pectin composition is essential for the normal growth and morphology pattern, as demonstrated by pectin-defective mutant phenotypes. Besides this basic role in plant physiology, pectin structure contributes to important biological processes affecting quality traits such as fruit firmness and ripening (de Godoy et al., 2013). Therefore, since in *Arabidopsis* mutants characterized by a significant reduction in the fucose content showed altered growth and cell wall composition (Reiter et al., 1993), the high fucose levels identified in the MC could be related to an increasing stability of cell wall components, improving the shelf life of treated sample.

Indeed, in *Arabidopsis* metabolic labeling experiment using sugar analogs compatible with click chemistry provided new insights into cell-wall structure and dynamics (Anderson et al., 2012). Using this approach, the authors found that an alkynylated fucose analog (FucAl) is metabolically incorporated into the cell walls of *Arabidopsis thaliana* roots and that a significant fraction of the incorporated FucAl is present in pectic rhamnogalacturonan (RG). FucAl-containing RG first localizes in cell walls as uniformly distributed that likely marks the sites of vescicle mediated delivery of new polysaccharides to growing cell walls.

The reduction of key metabolites of the primary metabolism during post-harvest stage is well known (Crisosto and Tonutti, 2013). This evidence was confirmed by the decrease of most of the amino acids analyzed after 7 days of post-harvest stage observed here in fig fruit. Among amino acids, phenylalanine was shown be significantly increased by EC in MC comparing to the untreated (CTR) at the same post-harvest stage. Phenylalanine is a key metabolite that leads to the beginning of the complex metabolic pathway of polyphenols, a very important category of secondary metabolism metabolites. Proteins involved in polyphenols biosynthesis and metabolism were shown be clearly affected during post-harvest stage in grape (Briz-Cid et al., 2016). Post-harvest fruits are very sensible to physiological modifications linked with senescence and cell membrane permeability. These phenotypic changes seemed to be induced by energy deficit (Zhang et al., 2016). Coating treatment with Ca^2+^ was effective in maintaining high energy status, increased hardness and antioxidant enzyme activities. It also reduced grape fruit softening and respiratory intensity (Zhang et al., 2016). These results obtained in grape agreed with our presented data in fig fruit. A higher level of some key anti-oxidant compounds was observed in EC treatment compared to untreated such as phenylalanine, benzoic acid, beta-sitosterol, and phenol-based metabolites. The compounds should actively contribute on the expected increase in antioxidant power of EC fruits.

The activity of the enzyme phenylalanine-ammonia –lyase has been shown to play an important role in the modulation of the accumulation of polyphenols in fruits (Martinelli and Tonutti, 2012). Indeed we may speculate that EC treatment should have a positive effect on polyphenol accumulation in the fruit with consequent beneficial effects at qualitative level. Treatments with chitosan solutions to mango fruits at post-harvest stage delayed climacteric peaks and affect soluble solid content as well titrable acidity, pH and sugar content (Cosme Silva et al., 2017). Our results confirmed that EC is useful in keeping fruit quality during cold storage and important qualitative fruit features. MC showed positive qualitative and antioxidant beneficial properties due to the general increase of organic acids and carbohydrates such as malic acid, malonic acid, glutamic acid, fucose, glucose-6-phosphate, and xylulose. The protective effect of coating was also demonstrated in reducing the attack of pathogens such as *Botrythis* sp. in pear fruit (Wang et al., 2017). This effect was due to the induction of defense-related enzyme. Simultaneously this treatment maintained nutritional value and reduced the loss of fruit weight. A 2% chitosan-based coating on fresh-cut melon was effective in maintaining quality attributes, promoting antioxidant metabolism, integrity of cell wall and membrane and increased soluble solids and carotenoids. These data agreed with our results that showed how EC was able to increase primary metabolism compounds such as amino acids and carbohydrates (Carvalho et al., 2016). Edible coating treatment delayed fruit senescence of jujube and it reduced the typical decrease in secondary metabolites (Kou et al., 2017). These evidences are in agreement with our data that highlighted how in MC some key metabolites belonging to the secondary metabolism were higher in amount compared with untreated fruits. The use of nano-bio-composite and thyme oil caused important changes on sweet cherry fruit decreasing fruit weight loss and increasing sugar content (Nabifarkhni et al., 2015) in accord with our data. Similar results were obtained with the use of EC in navel orange (Youssef et al., 2015).

Eventually, this metabolomics study highlighted that breba fig fruits exhibited some important changes in primary metabolism in response to EC treatment. This study showed how EC may have positive effects on the amount of important nutritional compounds of the primary metabolism. This post-harvest treatment induced some qualitative and quantitative improvements comparing with untreated fruit conditions after harvest. The analysis of principal components clearly highlighted that the primary metabolism of fruits treated by EC was clearly different from the one of untreated fruits. In addition EC treatment beneficially increased carbohydrate amounts and other key qualitative metabolites, such as beta-sitosterol and glycerol. Most of the amino acids were increased in abundance if EC was applied during post-harvest stage. EC had a clear and beneficial effect of mitigating the unavoidable reduction of aminoacid contents during cold storage. Among them, alanine, aspartic acid and glutamic acid were those aminoacids that were mostly affected by EC. These key primary metabolites may be considered as candidate biomarkers to test the effectiveness of this post-harvest treatment on maintaining high the quality of fruits at post-harvest stage. In addition, these changes pointed out the importance to analyze the metabolites in response to other post-harvest treatments. They may be included in preferable metabolic analysis to determine the physiological status of breba fig fruits after harvest. These metabolites may be analyzed with portable instruments recently appeared in the market for real time analysis of fruit qualitative and physiological conditions during shelf life. This study highlighted the ability of living fruit cells to modulate the amount of metabolites that may be manipulated with hormonal and environmental factors during post-harvest stage. These findings will enhance our understanding of the effects of post-harvest treatments such as EC on fruit chemical transformations and will improve our ability to maximize quality and minimize fruit losses.

## Author Contributions

AA and AG performed the experimental work. AA, PI, and FeM conceived the work. AA and AG conducted the statistical analysis. All authors contributed to wrote the paper.

## Conflict of Interest Statement

The authors declare that the research was conducted in the absence of any commercial or financial relationships that could be construed as a potential conflict of interest. The reviewer GL and handling Editor declared their shared affiliation at the time of the review.

## Footnotes

1 http://www.fao.org/faostat/en/#data/QC/visualize

2 https://cran.r-project.org/web/packages/Hmisc/index.html

3 https://cran.r-project.org/web/packages/PerformanceAnalytics/index.html

## Supplementary Material

FIGURE S1 Scatter plot matrix with the correlation coefficients between analyzed variables and their significance levels. The distribution of each variable is shown on the diagonal; the bivariate scatter plots with a fitted line are displayed on the bottom of the diagonal; the value of the correlation plus the significance level as stars was highlighted on the top of the diagonal. Asterisks indicate significance levels.

TABLE S1 List of the metabolites analysis with the metabolomic approach.
