# Supplementary figures and images for: Metabolic Profiling and Post-harvest Behavior of “Dottato” Fig (Ficus carica L.) Fruit Covered With an Edible Coating From O. ficus-indica
Source: Front Plant Sci. 2018 Sep 5;9:1321. doi: 10.3389/fpls.2018.01321 (PMC6134321; doi:10.3389/fpls.2018.01321)

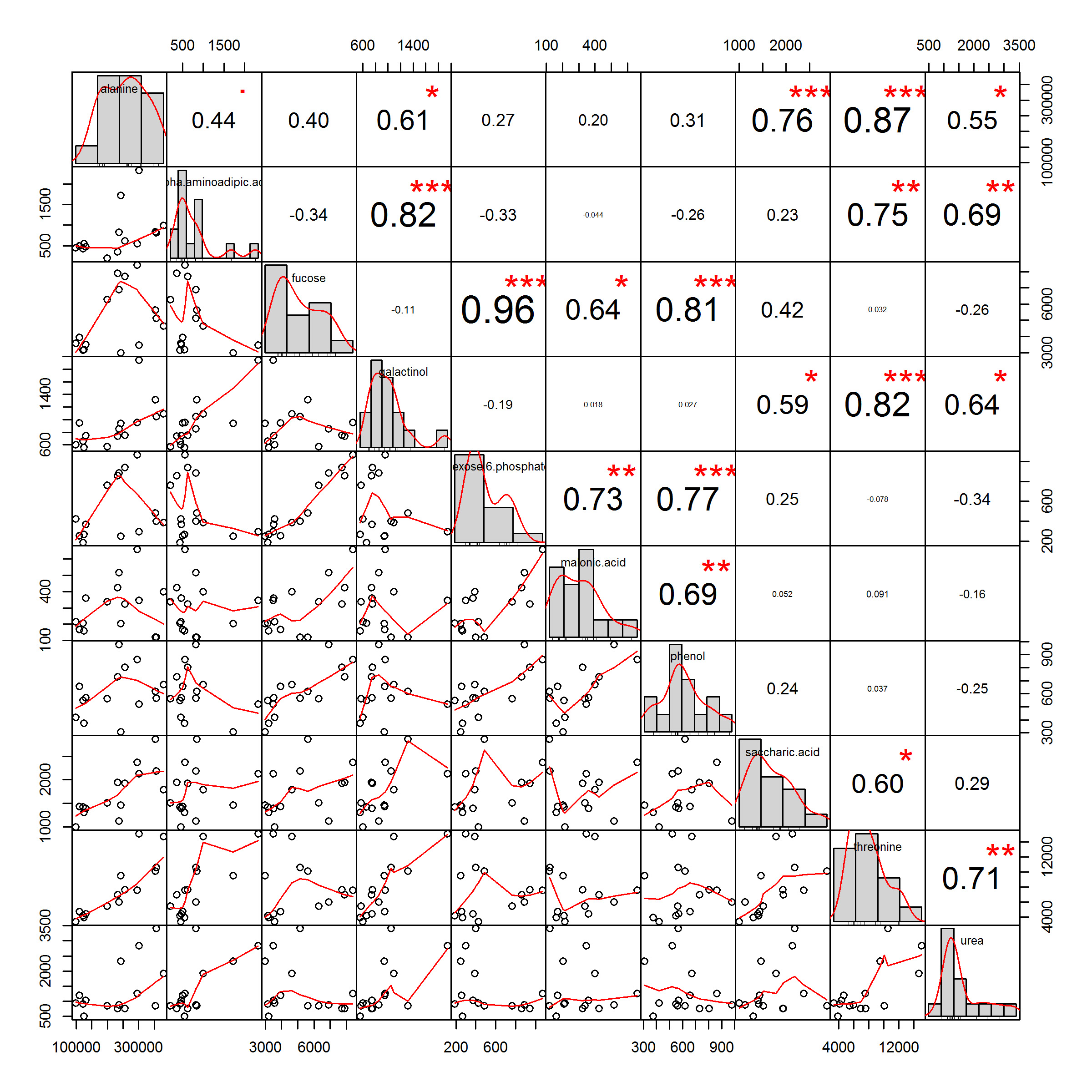

Supplement: FIGURE S1 — Scatter plot matrix with the correlation coefficients between analyzed variables and their significance levels. The distribution of each variable is shown on the diagonal; the bivariate scatter plots with a fitted line are displayed on the bottom of the diagonal; the value of the correlation plus the significance level as stars was highlighted on the top of the diagonal. Asterisks indicate significance levels. [file Image_1.JPEG]
